# Multiplex Dual-Target Reverse Transcription PCR for Subtyping Avian Influenza A(H5) Virus

**DOI:** 10.3201/eid3008.240785

**Published:** 2024-08

**Authors:** Malaya K. Sahoo, Ingrid E.A. Morante, ChunHong Huang, Daniel Solis, Fumiko Yamamoto, Uzoamaka C. Ohiri, Daniel Romero, Benjamin A. Pinsky

**Affiliations:** Stanford Health Care Clinical Virology Laboratory, Stanford, California, USA (M.K. Sahoo, I.E.A. Morante, U.C. Ohiri, D. Romero, B.A. Pinsky);; Stanford University School of Medicine, Stanford (C. Huang, D. Solis, F. Yamamoto, B.A. Pinsky)

**Keywords:** influenza, avian influenza, viruses, PCR, subtyping, H5, zoonoses, respiratory infections

## Abstract

An increased risk for human infection with avian influenza A(H5N1) viruses is of concern. We developed an internally controlled, dual-target reverse transcription PCR for influenza A(H5) subtyping. This test could be used to detect influenza A(H5) in clinical samples.

Highly pathogenic avian influenza (HPAI) viruses cause high mortality rates in wild birds and farmed poultry, and their potential for adaptation to humans remains a major pandemic threat (*1*). In 2021, a notable increase in avian influenza activity occurred, driven by the emergence of influenza A(H5N1) clade 2.3.4.4b viruses (*2*,*3*). Suspected mammal-to-mammal transmission during outbreaks among seals and minks led to concerns regarding the escalation of risk to humans (*4*–*7*). In 2024, the virus was identified in dairy cows in the United States (*8*), and exposure to infected cows has resulted in transmission to humans (*9*). To prepare for possible additional human cases, we developed an internally controlled, dual-target H5 subtyping quantitative reverse transcription PCR (qRT-PCR) based on primer–probe sequences from the World Health Organization (*10*). We evaluated this qRT-PCR by using synthesized nucleic acids, cultured avian and human virus isolates, and clinical upper respiratory specimens collected from patients with influenza A virus infection.

## The Study

We identified previously published primer–probe sets from the World Health Organization that target different regions of the influenza A(H5) hemagglutinin (HA) gene for combination into a dual-target H5 subtyping qRT-PCR (Appendix Table 1) (*10*). We introduced minor sequence changes to account for recent clade 2.3.4.4b diversity, and simultaneously, to limit primer–probe complexity (Table 1). We evaluated these primers and probes against complete North American H5 clade 2.3.4.4b HA gene sequences in the GISAID database (https://www.gisaid.org) during January 1, 2022–May 29, 2024, and found that 99.8% (5,975/5,987) of sequences aligned with a maximum of 1 mismatch to primer–probe set 1 and 97.7% (5,972/5,990) aligned with a maximum of 1 mismatch to primer–probe set 2 (Appendix Tables 2, 3). We observed no primer–probe mismatches compared with the influenza A H5 sequences obtained from persons with H5 infections in the United States (4 persons as of May 29, 2024) (Appendix Tables 4, 5). We combined the primer–probe sets in multiplex with primers–probes for the influenza A matrix (M) gene for pan–influenza A detection and with primers–probes for RNase P as an internal control (*11*).

**Table 1:** Primers and probes used in study of multiplex dual-target reverse transcription PCR for subtyping avian influenza A(H5) virus*

Target	Name	Sequence, 5′ → 3′	Final concentration
A(H5)	FluA_H5_v4_1F	TACCAGATACTGTCAATTTATTCAAC	400 nM
	FluA_H5_v4_1R	GTAACGACCCATTGGAGCACATCC	400 nM
	FluA_H5_v4_1Prb.FAM	FAM-CTGGCAATC/ZEN/ATG**R**T**R**GCTGGTCT-3IABkFQ	200 nM
		
	FluA_H5_v4_2F	TGGGTACCATCATAGCAATGAGCA	400 nM
	FluA_H5_v4_2R	AACTCCCTTCCAACTGCCTCAAA	400 nM
	FluA_H5_v4_2Prb.FAM	FAM-TGGGTACGC/ZEN/TGCGGACAAAGAATCCA-3IABkFQ	200 nM
A (M)	Pan-FluA-F	GACC**R**ATCCTGTCACCTCTGAC	400 nM
	Pan-FluA -R	AGGGCATT**Y**TGGACAAA**K**CGTCTA	400 nM
	Pan-FluA -prb_Q705	Q705-TGCAGTCCTCGCTCACTGGGCACG- BHQ-3	200 nM
Human RNase P	RNase P Fwd	AGATTTGGACCTGCGAGCG	100 nM
	RNase P Rev	GAGCGGCTGTCTCCACAAGT	100 nM
	RNase P Probe CF560	CF560-TTCTGACCTGAAGGCTCTGCGCG-BHQ-1	50 nM

*H5 probes were purchased from Integrated DNA Technologies (https://www.idtdna.com). The probes contain the internal quencher, ZEN, in addition to the 3′ Iowa BHQ FQ (3IABkFQ) (proprietary to Integrated DNA Technologies). Influenza A (M) and human RNase P probes were purchased from Biosearch Technologies (https://www.biosearchtech.com). Bold type indicates mixed bases. BHQ, Black Hole Quencher; CF560, CalFluor 560; FAM, 6-carboxyfluorescein; M, matrix gene; Q705, Quasar 705.

We determined the 95% lower limit of detection (LLOD) by using single-stranded DNA (ssDNA) encoding the H5 target sequences from HPAI virus clade 1 (GenBank accession no. JQ966928, A/condor/Guangdong/139/2003 [H5N1]) (*12*) and clade 2.3.4.4b (GenBank accession no. OP499866, A/red-tailed hawk/Kansas/W22–198/2022 [H5N1]) viruses (Appendix Table 6). The primer–probe target regions of this clade 2.3.4.4b H5 sequence are identical to the recent H5 sequences from dairy cows and humans. We combined the 2 clade 1 ssDNA targets at equal copy numbers; we did the same with the clade 2.3.4.4b ssDNA targets. We also combined these H5 clade mixes at equal copy numbers with ssDNA encoding the pan–influenza A M gene target. We then diluted the clade 1 ssDNA mix and clade 2.3.4.4b ssDNA mix to 2, 1, and 0.5 copies/μL in pooled influenza A–negative eluates extracted from clinical upper respiratory specimens. We then performed the H5 subtyping qRT-PCR (Appendix). We tested 20 replicates at each level for both mixes. The 95% LLOD for the H5 target was 2.5 copies/μL (95% CI 1.8–5.3 copies/μL) for the clade 1 ssDNA mix and <0.5 copies/μL (we were unable to calculate 95% CI) for the clade 2.3.4.4b ssDNA mix.

We also evaluated the H5 subtyping multiplex qRT-PCR by using genomic RNA from a clade EurAsian non-goose/GuangDong (Gs/Gd) isolate, Kilbourne F181, A/duck/Singapore/645/1997 (H5N3). We assigned this genomic RNA an estimated concentration in copies/μL by using the clade 2.3.4.4b ssDNA as standard curve, then diluted it to 2, 1, and 0.5 copies/μL in pooled influenza A–negative eluates. We tested 20 replicates at each level. The 95% LLOD for the H5 target was 0.6 copies/μL (95% CI 0.5–0.7 copies/μL). In addition, we tested 10-fold serial dilutions from 4.6 to 0.6 log_10_ copies/mL of the Kilborne F181 genomic RNA in duplicate (Appendix Figure), which indicated 0.97 reaction efficiency for the H5 target.

We then evaluated the H5 subtyping qRT-PCR by using genomic RNA from additional H5 viruses, including 5 low pathogenicity avian influenza non-Gs/Gd isolates from the United States (Table 2). We tested each eluate in duplicate. Although we detected H5 RNA in all reactions, the difference in mean cycle threshold (Ct) values between the H5 and pan–influenza A (M gene) targets varied widely (range 0.6–9.9 cycles). Testing the H5 primer–probe sets individually revealed that the US non-Gs/Gd genomic RNAs were detected only by primer–probe set 1. The A/mallard/Minnesota/16-041335-3/2016 (H5N2) eluate showed the greatest Ct difference, consistent with the highest number of H5 primer–probe mismatches in primer–probe set 1 (9 mismatches) (Appendix Tables 5, 6).

**Table 2:** Avian influenza A(H5) virus genomic RNA used to evaluate inclusivity in study of multiplex dual-target reverse transcription PCR for subtyping avian influenza A(H5) virus*

Genome	Clade	GenBank accession no.	H5 Ct	M Ct	Ct difference
Kilbourne F181, A/duck/Singapore/645/1997 (H5N3)	EA-non-Gs/Gd	NA	18.3	17.7	0.6
A/quail/California/14-012546-1/2014 (H5N8)	Am-non-Gs/Gd	NA	22.9	19.0	3.9
A/mallard/Minnesota/16-041335-3/2016 (H5N2)	Am-non-Gs/Gd	MH546659	32.1	22.3	9.9
A/emperor goose/Alaska/17-004479-1/2016 (H5N2)	Am-non-Gs/Gd	MH546451	26.8	23.3	3.6
A/glaucous-winged gull/Alaska/16-041335-19/2016 (H5N2)	Am-non-Gs/Gd	MH546475	23.3	21.8	1.5
A/northern pintail/Alaska/16-041335-5/2016 (H5N2)	Am-non-Gs/Gd	MH546883	20.9	19.1	1.9

*Kilbourne F181 genomic RNA was obtained from BEI Resources (https://www.beiresources.org). Am-non-Gs/GD A(H5) genomic RNA was obtained from the US Department of Agriculture. The genomic RNA from these influenza A(H5) viruses was diluted 1:10 buffer AVE plus carrier RNA (QIAGEN, https://www.qiagen.com) and tested in duplicate. Ct values are means. Am-non-Gs/Gd, non-goose/GuangDong from the United States; Ct, cycle threshold; EA-non-Gs/Gd, Eurasian non-goose/GuangDong; M, matrix gene; NA, not available.

To determine specificity, we tested genomic RNA from cultured human and avian virus isolates encoding non-H5 HA genes (Table 3). We detected the pan–influenza A target in all eluates; median Ct value was 17.3 (range 13.6–20.7). We did not detect the H5 target in any eluates.

**Table 3:** Non-H5 human and avian influenza A virus genomic RNA used to evaluate H5 specificity in study of multiplex dual-target reverse transcription PCR for subtyping avian influenza A(H5) virus*

HA gene	Genome	BEI catalog no.
H1	A/Brisbane/59/2007 (H1N1)	NR-20080
H1 pdm09	A/California/04/2009 (H1N1)pdm09	NR-14689
H2	A/duck/Germany/1215/1973 (H2N3)	NR-2762
H3	A/Brisbane/10/2007 (H3N2)	NR-20081
H4	A/duck/Czechoslovakia/1956 (H4N6)	NR-43012
H6	A/shearwater/Australia/1/1973 (H6N5)	NR-43014
H8	A/turkey/Ontario/6118/1968 (H8N4)	NR-43015
H9	A/turkey/Wisconsin/1/1966 (H9N2)	NR-43016
H10	A/chicken/Germany/N/1949 (H10N7)	NR-2765
H11	A/duck/Memphis/546/1974 (H11N9)	NR-43017
H12	A/duck/Alberta/60/1976 (H12N5)	NR-43018
H13	A/gull/Maryland/704/1977 (H13N6)	NR-43019
H14	A/mallard/Astrakhan/263/1982 (H14N5)	NR-43020
H15	A/shearwater/Australia/2576/1979 (H15N9)	NR-43021
H16	A/shorebird/Delaware/172/2006 (H16N3)	NR-43022

*All genomic RNA obtained from BEI Resources (https://www.beiresources.org). The genomic RNA from these influenza A (H5) viruses was diluted 1:10 in buffer AVE plus carrier RNA (QIAGEN, https://www.qiagen.com). HA, hemagglutinin; pdm09, pandemic 2009.

We performed further specificity experiments by using residual upper respiratory swab samples in viral transport media submitted for clinical respiratory virus testing. We conducted this work with Stanford Institutional Review Board approval (protocol no. 68234); individual consent was waived. We evaluated 100 samples collected during the 2022–2023 respiratory virus season that were positive for influenza A by using routine clinical methods (98 by using Hologic Panther Fusion [Hologic, https://www.hologic.com] and 2 by using Cepheid GeneXpert [Cepheid, https://www.cepheid.com]), including 29 known co-infections (13 respiratory syncytial virus [RSV], 7 rhinovirus, 3 adenovirus, 3 human metapneumovirus, 1 parainfluenza-3, 1 parainfluenza 4, and 1 with both RSV and rhinovirus). In addition, we tested 55 upper respiratory samples collected in April 2024 that were negative for influenza A, influenza B, RSV, parainfluenza virus 1–4, rhinovirus, adenovirus, and human metapneumovirus on the Panther Fusion by using the H5 subtyping qRT-PCR. We extracted total nucleic acids from 400 µL and eluted them in 60 µL buffer AVE on the BioRobot EZ1 (Qiagen) by using the EZ1 virus mini kit 2.0, according to the manufacturer’s recommendations. We detected the pan–influenza A target in 97.0% (97/100) of the influenza A–positive samples; median Ct value was 24.1 (range 12.6–37.7). All 3 of the samples that were not detected in the pan–influenza A channel of the H5 multiplex had detectable RNase P (Ct values of 22.5, 18.7, and 20.8), indicating adequate extraction and the absence of inhibitors. The original influenza A Ct values (Panther Fusion) for those 3 samples were 41.4, 38.0, and 38.2. Upon repeat testing, influenza A RNA was detected only in the sample with the lowest original Ct value for influenza A (C_t_ 34.8) by the H5 multiplex. None of those influenza A–positive samples had detectable H5 RNA. Of the respiratory virus panel negative samples, all had detectable RNase P; median Ct value was 23.9 (range 17.7–29.8). None of the panel negative samples had detectable H5 RNA.

## Conclusions

HPAI H5 viruses, particularly clade 2.3.4.4b viruses, pose an emerging pandemic threat. This internally controlled dual-target influenza A(H5) qRT-PCR demonstrated high analytical performance and could be used to directly test samples from patients with suspected HPAI A(H5) virus infection or as a secondary test to subtype known influenza A–positive samples after routine respiratory virus testing. The dual-target design reduces the likelihood of false negatives, as evidenced by detection of low pathogenicity avian influenza H5 viruses with substantial target mismatch. However, continuous sequence surveillance and updating of the primer–probe sets will be required to ensure that this assay accounts for ongoing viral evolution (*13*).

Appendix Additional information about multiplex dual-target reverse transcription PCR for subtyping avian influenza A(H5) virus.

## References

[1] Morens DM, Park J, Taubenberger JK. Many potential pathways to future pandemic influenza. Sci Transl Med. 2023;15:eadj2379. 10.1126/scitranslmed.adj2379 37851826

[2] Sun Y, Zhang T, Zhao X, Qian J, Jiang M, Jia M, et al. High activity levels of avian influenza upwards 2018-2022: A global epidemiological overview of fowl and human infections. One Health. 2023;16:100511. 10.1016/j.onehlt.2023.100511 37363261 PMC10288038

[3] Szablewski CM, Iwamoto C, Olsen SJ, Greene CM, Duca LM, Davis CT, et al. Reported global avian influenza detections among humans and animals during 2013–2022: comprehensive review and analysis of available surveillance data. JMIR Public Health Surveill. 2023;9:e46383. 10.2196/46383 37651182 PMC10502594

[4] Kniss K, Sumner KM, Tastad KJ, Lewis NM, Jansen L, Julian D, et al. Risk for infection in humans after exposure to birds infected with highly pathogenic avian influenza A(H5N1) virus, United States, 2022. Emerg Infect Dis. 2023;29:1215–9. 10.3201/eid2906.230103 37095080 PMC10202859

[5] Gilbertson B, Subbarao K. Mammalian infections with highly pathogenic avian influenza viruses renew concerns of pandemic potential. J Exp Med. 2023;220:e20230447. 10.1084/jem.20230447 37326966 PMC10276204

[6] Puryear W, Sawatzki K, Hill N, Foss A, Stone JJ, Doughty L, et al. Highly pathogenic avian influenza A(H5N1) virus outbreak in New England seals, United States. Emerg Infect Dis. 2023;29:786–91. 10.3201/eid2904.221538 36958010 PMC10045683

[7] Agüero M, Monne I, Sánchez A, Zecchin B, Fusaro A, Ruano MJ, et al. Highly pathogenic avian influenza A(H5N1) virus infection in farmed minks, Spain, October 2022. Euro Surveill. 2023;28:2300001. 10.2807/1560-7917.ES.2023.28.3.2300001 36695488 PMC9853945

[8] Burrough ER, Magstadt DR, Petersen B, Timmermans SJ, Gauger PC, Zhang J, et al. Highly pathogenic avian influenza A(H5N1) clade 2.3.4.4b virus infection in domestic dairy cattle and cats, United States, 2024. Emerg Infect Dis. 2024;30:1335–43. 10.3201/eid3007.240508 38683888 PMC11210653

[9] Uyeki TM, Milton S, Abdul Hamid C, Reinoso Webb C, Presley SM, Shetty V, et al. Highly pathogenic avian influenza A(H5N1) virus infection in a dairy farm worker. N Engl J Med. 2024;390:2028–9. 10.1056/NEJMc2405371 38700506

[10] World Health Organization. WHO information for molecular diagnosis of influenza virus—update. 2021 Feb 7 [cited 2023 Jun 25]. https://www.who.int/teams/global-influenza-programme/laboratory-network/quality-assurance/eqa-project/information-for-molecular-diagnosis-of-influenza-virus

[11] Huang C, Sahoo MK, Verghese M, Sibai M, Solis D, Mfuh KO, et al. Interepidemic respiratory syncytial virus during the COVID-19 pandemic. Microbiol Spectr. 2022;10:e0094722. 10.1128/spectrum.00947-22 35467362 PMC9241888

[12] Jiao P, Yuan R, Song Y, Wei L, Ren T, Liao M, et al. Full genome sequence of a recombinant H5N1 influenza virus from a condor in southern China. J Virol. 2012;86:7722–3. 10.1128/JVI.01043-12 22733885 PMC3416285

[13] Kandeil A, Patton C, Jones JC, Jeevan T, Harrington WN, Trifkovic S, et al. Rapid evolution of A(H5N1) influenza viruses after intercontinental spread to North America. Nat Commun. 2023;14:3082. 10.1038/s41467-023-38415-7 37248261 PMC10227026

