# Supplementary material for: Multiplex Dual-Target Reverse Transcription PCR for Subtyping Avian Influenza A(H5) Virus
Source: Emerg Infect Dis. 2024 Aug;30(8):1710–3. doi: 10.3201/eid3008.240785 (PMC11286043; doi:10.3201/eid3008.240785)
Supplement: Appendix — Additional information about multiplex dual-target reverse transcription PCR for subtyping avian influenza A(H5) virus. [file 24-0785-Techapp-s1.pdf]

Article DOI: https://doi.org/10.3201/eid3008.240785

EID cannot ensure accessibility for supplementary materials supplied by authors. Readers who have difficulty accessing supplementary content should contact the authors for assistance.

Multiplex Dual-Target Reverse Transcription PCR for Subtyping Avian Influenza A(H5) Virus

Appendix

Methods

We performed reverse transcription quatitative PCR (RT-qPCR) by using the Invitrogen Superscript III Platinum One-Step qRT-PCR kit (Thermo Fisher Scientific, https://www.thermofisher.com) on the Rotor-Gene Q real-time PCR instrument (QIAGEN, https://www.qiagen.com). Each 25-µL reaction contained 12.5 µL of 2X buffer, 0.5 µL of enzyme mix, 2 µL of primer–probe mix, and 10 µL of nucleic acids. Cycling conditions were as follows: hold at 52°C for 15 min, 94°C for 2 min, then 45 cycles of 94°C for 15 sec, 55°C for 40 sec, and 68°C for 20 sec. We analyzed the fluorescence signal in the green (H5), crimson (pan-influenza A), and yellow (RNase P) channels. We set thresholds at 0.1 normalized fluorescence units for all targets. We interpreted any exponential amplification curve crossing the green channel as subtype H5. The 95% lower limit of detection was determined in R (https://www.r-project.org) by using probit regression.

Appendix Table 1. Original Influenza A(H5) Primer and Probe Sequences

Name	Sequence (5′→3′)	Source	Notes
H5.2344-1673F	TACCAAATA**Y**TGTCAATTTATTCAAC	School of Public Health, Hong Kong University, Hong Kong SAR, China	A (H5) clade 2.3.4.4
H5.2344-1749R	GTAA**Y**GACCC**R**TT**R**GA**R**CACATCC		
H5.2344-1718Prb_FAM	CTGGCAATCAT**DR**TGGCTGGTCT		
H5-1012F	TGGGTACCACCATAGCAATGAGCA	Centre for Health Protection, Hong Kong SAR, China	A (H5)
H5-1155R	AATTCCCTTCCAACGGCCTCAAAC		
H5-1042P-P2_CFO560	TGGGTACGCTGCAGACAAAGAATCCA		

SAR, Special Administrative Region. Mixed bases are in bold

Appendix Table 2. Alignment of Primer/Probe Set 1 to North American Influenza A(H5) Hemagglutinin (HA) Sequences available from GISAID January 1, 2022 to May 29, 2024.

	Forward Primer	Probe	Reverse Primer*		
Reference	TACCAGATACTGTCAATTTATTCAAC	CTGGCAATCATGATGGCTGGTCT	GGATGTGCTCCAATGGGTCGTTAC		
Set 1	TACCAGATACTGTCAATTTATTCAAC	CTGGCAATCATG**R**T**R**GCTGGTCT	GGATGTGCTCCAATGGGTCGTTAC	No.	%
	....	....	....	5529	92.4
	.......................C..	....	....	82	1.4
	.........T................	....	....	58	1.0
	....	....	......................G.	44	0.7
	....	............G..........	....	39	0.7
	....	....	.......T................	36	0.6
	.....A....................	....	....	25	0.4
	..T.......................	....	....	24	0.4
	....	....	...................A....	21	0.4
	....	..............A........	....	13	0.2
	....	...............T.......	....	11	0.2
	....	...........A...........	....	8	0.1
	....	............T..........	....	8	0.1
	....	....	................A.......	8	0.1
	.................C........	....	....	7	0.1
	....	.......G...............	....	6	0.1
	....	....................C..	....	6	0.1
	....	.........G.............	....	5	0.1
	....	...........T...........	....	5	0.1
	....	....	..........T.............	4	0.1
	....	....	................T.......	4	0.1
	...........A..............	....	....	4	0.1
	....	....	............T...........	4	0.1
	....	T......................	....	4	0.1
	.........T................	............T..........	....	3	0.1

*The reverse primer sequence is presented as its reverse complement.
Sequences accessed May 29, 2024. Aligned sequences: 5987. GenBank: PP577943/GISAID: EPI3171488 (A/Texas/37/2024) is used as reference.
Primer and probe alignments are in-phase. Top 25 most abundant sequences are listed. Mixed bases are in bold.

Appendix Table 3. Alignment of Primer/Probe Set 2 to North American Influenza A(H5) Hemagglutinin (HA) Sequences available from GISAID January 1, 2022 to May 29, 2024.

	Forward Primer	Probe	Reverse Primer*		
Reference	TGGGTACCATCATAGCAATGAGCA	TGGGTACGCTGCGGACAAAGAATCCA	TTTGAGGCAGTTGGAAGGGAGTT		
Set 2	TGGGTACCATCATAGCAATGAGCA	TGGGTACGCTGCGGACAAAGAATCCA	TTTGAGGCAGTTGGAAGGGAGTT	No.	%
	....	....	....	5085	84.9
	....	............A.............	....	209	3.5
	......T.................	....	....	93	1.6
	...A....................	....	....	74	1.2
	....	...............T..........	....	48	0.8
	....	....	..............C........	45	0.8
	.........C..............	............A.............	....	43	0.7
	....	......T...................	....	41	0.7
	....	...A......................	....	32	0.5
	...............T........	....	....	26	0.4
	....	....	.....A.................	17	0.3
	......T.................	..................G.......	....	15	0.3
	...T....................	....	....	15	0.3
	....	............T.............	....	14	0.2
	....	....	.................A.....	13	0.2
	....	....	..C....................	12	0.2
	....	....	........T..............	12	0.2
	....	............A.....G.......	....	10	0.2
	.........C..............	....	....	9	0.2
	..............A.........	....	....	9	0.2
	....	....	....................A..	9	0.2
	....	...T......................	....	8	0.1
	....	..................G.......	....	8	0.1
	.....................A..	....	....	8	0.1
	....	....	..............G........	8	0.1

*The reverse primer sequence is presented as its reverse complement.
Sequences accessed May 29, 2024. Aligned sequences: 5990. GenBank: PP577943/GISAID: EPI3171488 (A/Texas/37/2024) is used as reference.
Primer and probe alignments are in-phase. Top 25 most abundant sequences are listed.

Appendix Table 4. Alignment of Primer/Probe Set 1 to Avian Influenza A(H5) Sequences from Human Cases and Dairy Cows.

	Forward Primer	Probe	Reverse Primer*
Reference	TACCAGATACTGTCAATTTATTCAAC	CTGGCAATCATGATGGCTGGTCT	GGATGTGCTCCAATGGGTCGTTAC
Set 1	TACCAGATACTGTCAATTTATTCAAC	CTGGCAATCATGRTRGCTGGTCT	GGATGTGCTCCAATGGGTCGTTAC
Human: A/Texas/37/2024	....	....	....
Human: A/Michigan/90/2024	....	....	....
Human: A/Michigan/91/2024	....	....	....
Human: A/Colorado/18/2022	....	....	....
A/dairy cow/Texas/24_009367–003/2024	....	.......G...............	....
A/dairy cow/Texas/24_009367–005/2024	....	.......G...............	....
A/dairy cow/Texas/24_009367–009/2024	....	.......G...............	....
A/dairy cow/Texas/24_009367–010/2024	....	.......G...............	....
A/dairy cow/Texas/24_009367–013/2024	....	.......G...............	....
A/dairy cow/Texas/24_009367–014/2024	....	.......G...............	....
Other 190 sequences from dairy cows	....	....	....

*The reverse primer sequence is presented as its reverse complement.
GenBank: PP577943/GISAID: EPI3171488 (A/Texas/37/2024) is used as reference. Mixed bases are in bold. Primer and probe alignments are in-phase.
Note: The 6 dairy cow sequences showing a mismatch with primer/probe set 1 do not contain any mismatches with primer/probe set 2.

Appendix Table 5. Alignment of Primer/Probe Set 2 to Avian Influenza A(H5) Sequences from Human Cases and Dairy Cows.

	Forward Primer	Probe	Reverse Primer*
Reference	TGGGTACCATCATAGCAATGAGCA	TGGGTACGCTGCGGACAAAGAATCCA	TTTGAGGCAGTTGGAAGGGAGTT
Set 2	TGGGTACCATCATAGCAATGAGCA	TGGGTACGCTGCGGACAAAGAATCCA	TTTGAGGCAGTTGGAAGGGAGTT
Human: A/Texas/37/2024	....	....	....
Human: A/Michigan/90/2024	....	....	....
Human: A/Michigan/91/2024	....	....	....
Human: A/Colorado/18/2022	....	....	....
A/dairy cow/Texas/24_009110–003/2024	....	...A......................	....
A/dairy cow/South Dakota/24_010354–001/2024	....	...A......................	....
A/dairy cow/Texas/24_009776–002/2024	...........T............	....	....
Other 193 sequences from dairy cows	....	....	....

*The reverse primer sequence is presented as its reverse complement.
GenBank: PP577943/GISAID: EPI3171488 (A/Texas/37/2024) is used as reference.Primer and probe alignments are in-phase.
Note: The 3 dairy cow sequences showing a mismatch with primer/probe set 2 do not contain any mismatches with primer/probe set 1.

Appendix Table 6. Single-Stranded DNA Sequences

Name	H5 Target Sequence (5′→3′)	GenBank Reference	Location
ssD_FluA_H5_T1C1	GTATGACTACCCGCAGTATTCAGAAGAAGCAAGACTAAAAAGAGAGGAAATAAGTGGAGTAAAATTGGAATCAATAGGAACTTACCA**A**ATACTGTCAATTTATTC**T**ACAGTGGCGAGTTCCCTAGCACTGGCAATCATGGTAGCTGGTCTATCTTTATGGATGTGCTCCAATGGGTCGTTACAATGCAGAAT	JQ966928	1534–1725
ssD_FluA_H5_T2C1	ATGGTTGGTATGGGTACCA**C**CATAGCAATGA**A**CAGGGGAGTGGGTACGCTGC**A**GACAAAGAATCCACTCAAAAGGCAATAGATGGAGTCACCAATAAGGTCAACTCGATCATTGACAAAATGAACACTCAGTTTGAGGC**C**GTTGGAAGGGA**A**TTTAATAACTTA	JQ966928:	1122–1285
ssD_FluA_H5_T1C2	AGTAGGAACTTACCAGATACTGTCAATTTATTCAACAGCGGCAAGTTCCCTAGCACTGGCAATCATGATGGCTGGTCTATCTTTATGGATGTGCTCCAATGGGTCGTTACAGTGCAGAAT	OP499866	1591–1710
ssD_FluA_H5_T2C2	ATGGTTGGTATGGGTACCATCATAGCAATGAGCAGGGGAGTGGGTACGCTGCGGACAAAGAATCCACCCAAAAGGCAATAGATGGAGTTACCAATAAGGTCAACTCAATCATTGACAAAATGAACACTCAATTTGAGGCAGTTGGAAGGGAGTTTAATAACTTA	OP499866	1107−1270

Primer and probe targets are underlined. Mismatched bases are in bold. Single-stranded DNA was synthesized by Integrated DNA Technologies.

Appendix Table 7. Alignment of Primer/Probe Set 1 to Avian Influenza A (H5) American-non Goose GuangDong Sequences

	Forward Primer	Probe	Reverse Primer*
Reference	TACCAGATACTGTCAATTTATTCAAC	CTGGCAATCATGATGGCTGGTCT	GGATGTGCTCCAATGGGTCGTTAC
**Set 1**	TACCAGATACTGTCAATTTATTCAAC	CTGGCAATCATG**R**T**R**GCTGGTCT	GGATGTGCTCCAATGGGTCGTTAC
A/mallard/Minnesota/16–041335–3/2016 (H5N2)	..T......T.A.....C..CG....	..............A........	.......T...........A..G.
A/emperor goose/Alaska/17–004479–1/2016 (H5N2)	..T........A.....C..C.....	..............A........	................A..A..G.
A/glaucous-winged gull/Alaska/16–041335–19/2016 (H5N2)	..T........A.....C........	..............A........	................A..A..G.
A/northern pintail/Alaska/16–041335–5/2016 (H5N2)	..T........A.....C........	..............A........	................A..A..G.

*The reverse primer sequence is presented as its reverse complement.
GenBank: PP577943/GISAID: EPI3171488 (A/Texas/37/2024) is used as reference. Mixed bases are in bold.

Appendix Table 8. Alignment of Primer/Probe Set 2 to Avian Influenza A(H5) American-non Goose GuangDong Sequences

	Forward Primer	Probe	Reverse Primer*
Reference	TGGGTACCATCATAGCAATGAGCA	TGGGTACGCTGCGGACAAAGAATCCA	TTTGAGGCAGTTGGAAGGGAGTT
**Set 2**	TGGGTACCATCATAGCAATGAGCA	TGGGTACGCTGCGGACAAAGAATCCA	TTTGAGGCAGTTGGAAGGGAGTT
A/mallard/Minnesota/16–041335–3/2016 (H5N2)	C.......................	...A..T.....A...........T.	..C..A..T.....G.AA..A..
A/emperor goose/Alaska/17–004479–1/2016 (H5N2)	C..A....................	...A..T.....A...........T.	..C..A..T.......AA..A..
A/glaucous-winged gull/Alaska/16–041335–19/2016 (H5N2)	C..A....................	...A..T.....A...........T.	..C..A..C.....G.AA..A..
A/northern pintail/Alaska/16–041335–5/2016 (H5N2)	C..A....................	...A..T.....A...........T.	..C..A..C.....G.AA..A..

*The reverse primer sequence is presented as its reverse complement.
GenBank: PP577943/GISAID: EPI3171488 (A/Texas/37/2024) is used as reference. Primer and probe alignments are in-phase.

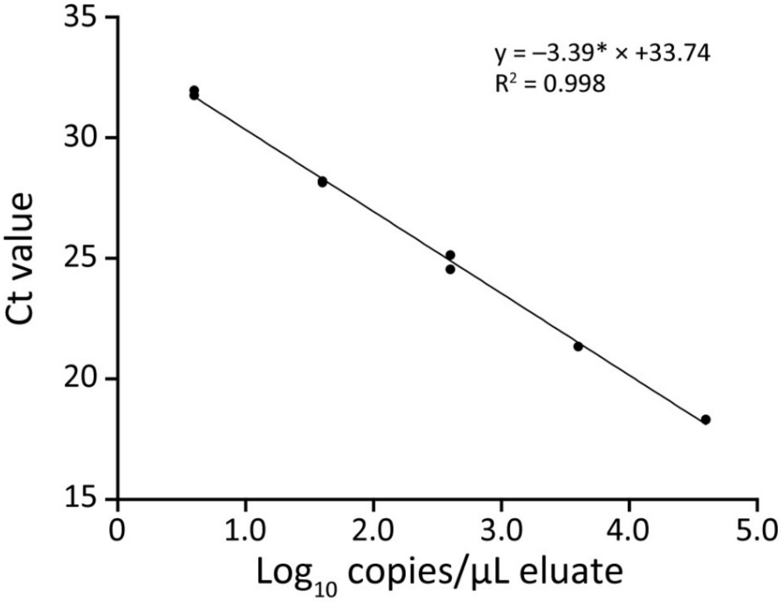

Appendix Figure. Ten-fold serial dilutions of Kilbourne F181, A/duck/Singapore/645/1997 (H5N3) (BEI Resources Catalog # NR-9682) genomic RNA from 4.6 to 0.6 log_{10} copies/mL were tested in duplicate using the H5 subtyping multiplex RT-qPCR. Filled circles, individual replicates. Solid line, simple linear regression (GraphPad Prism 10 for macOS, version 10.2.3).
